# Effect of fingolimod therapy on quantitative macular changes among patients with relapsing-remitting multiple sclerosis: a four-year follow-up study from Oman

**DOI:** 10.1186/s12886-022-02701-7

**Published:** 2022-12-05

**Authors:** Aseel A. Al-Rashdi, Buthaina I. Sabt, Abdullah S. Al-Mujaini

**Affiliations:** 1 Oman Medical Specialty Board, Muscat, Oman; 2 Department of Ophthalmology, Sultan Qaboos University Hospital, Muscat, Oman; 3 Department of Ophthalmology, College of Medicine and Health Sciences, Sultan Qaboos University, Muscat, Oman

**Keywords:** Relapsing remitting multiple sclerosis, Fingolimod therapy, Spectral-domain optical coherence tomography, Central foveal thickness, Total macular volume

## Abstract

**Purpose:**

Fingolimod (FTY-720) is an immunomodulatory oral agent approved for the treatment of relapsing-remitting multiple sclerosis (RRMS); however, several clinical trials have shown that some recipients may develop macular oedema (ME) as an adverse reaction. As there are no studies assessing the long-term (> 1 year) effect of fingolimod on the macula, this study aimed to evaluate the quantitative effect of fingolimod therapy on central macular thickness (CMT) and total macular volume (TMV) over a four-year period.

**Methods:**

This retrospective longitudinal cohort study was performed between January 2014 and December 2018. A total of 21 patients with RRMS receiving fingolimod therapy were recruited and followed-up over 4 years to assess CMT and TMV changes measured using spectral domain optical coherence tomography. A paired sample t-test was used to compare mean CMT and TMV values calculated at baseline prior to the initiation of fingolimod therapy with those observed at three, six, 12, 24, 36 and 48 months of treatment.

**Results:**

None of the patients developed ME over the four-year study period. In addition, there was no significant difference in baseline mean CMT values and those observed at a four-year follow-up. Although mean TMV values remained constant initially, there was a significant decrease towards the end of the study period.

**Conclusions:**

Long-term fingolimod therapy did not result in significant CFT changes. While there was a reduction in TMV towards the end of the study, this is likely due to the degenerative effect of the disease itself on the nerve fibres of the retina.

## Introduction

Multiple sclerosis (MS) is a neurodegenerative disease of the central nervous system (CNS) which results from immune-mediated inflammation and demyelination of the axons [1]. Symptoms vary according to the site of the lesions. In total, MS has four subtypes based on its clinical course: relapsing-remitting (RRMS), secondary progressive, primary progressive and progressive-relapsing MS [1]. As there is currently no cure for MS, the goal of medical management is to halt the pathophysiological progression of the disease. Fingolimod (FTY-720) is the first immunomodulatory oral agent approved by the U.S. Food and Drug Administration for the treatment of relapsing forms of MS. It is a structural analogue to sphingosine-1-phosphate (S1P), working as a receptor modulator [2]. It acts by inhibiting the emergence of lymphocytes from the lymph nodes, reducing infiltration to the CNS.

Two clinical phase III trials, the 12-month Trial Assessing Injectable Interferon vs FTY720 Oral in RRMS (TRANSFORMS) and the 24-month FTY720 Research Evaluating Effects of Daily Oral Therapy in MS (FREEDOMS), have demonstrated reduced disease activity and relapse rates with the administration of oral fingolimod therapy in the treatment of MS compared to interferon β-1a and a placebo [3, 4]. However, teratogenicity leading to abnormal foetal development, including acrania and teratology of Fallot, remains a major drawback of this medication; in a relatively small cohort study of 66 pregnancies, Karlsson et al. reported the frequency of teratogenicity to be 7.6% [5]. In addition, macular oedema (ME) was reported as an adverse reaction in several recipients of fingolimod in the aforementioned trials [3, 4]. Most of these cases occurred within 3–4 months of initiation of treatment and were diagnosed by clinical examination and assessment of macular thickness using spectral domain optical coherence tomography (SD-OCT).

Fingolimod-associated ME (FAME) appears to be dose-dependent; in renal transplant patients, a daily therapeutic dose of 0.5 mg has been found to result in an incidence range of 0–2.08%, with a cumulative mean (CM) of 0.82%, whereas a dose of 1.25 mg has been found to result in an incidence range of 0–1.63%, with a CM of 1.23% [6–10]. In a recent population-based study of RRMS patients, the final incidence rate of FAME was 1.32% (*n* = 3/228 patients) over a six-year follow-up period at a 95% confidence interval (CI) [11]. Surprisingly, in one of these cases, the effect occurred bilaterally 21 months after starting treatment. It is worth mentioning that this patient had taken interferon β-1a for 2 years prior to starting fingolimod and was on inhaled salbutamol for asthma [11].

In a five-month observational study, SD-OCT demonstrated macular volume changes among 30 consecutive patients with MS receiving fingolimod compared to a reference group of 30 patients with MS who had never been exposed to the medication [12]. It was found that 74% of eyes in the fingolimod-treated group exhibited an increase in macular volume versus 37% of eyes in the comparison group. Another recent study of 23 RRMS patients receiving fingolimod treatment over 12 months showed no significant quantitative changes in terms of either central foveal thickness (CFT) or total macular volume (TMV) [13]. However, to the best of the authors’ knowledge, no studies have yet shown the long-term (> 1 year) macular impact of fingolimod in RRMS patients. As such, our study aimed to evaluate the long-term quantitative effect of fingolimod therapy on the macula of RRMS patients over a four-year period using SD-OCT.

## Methods

We conducted a retrospective, longitudinal cohort study between January 2014 and December 2018. During this period, a total of 27 patients were diagnosed with RRMS according to the revised McDonald criteria [1]. Patients were initially followed up at the neurology clinic of the Sultan Qaboos University Hospital (SQUH), a tertiary hospital in Muscat, the capital of Oman. Fingolimod therapy was initiated for all patients as a second-line therapy after failure to respond to first-line treatment prescribed by a neurologist. All of the patients fulfilled the fingolimod treatment criteria using the approved therapeutic dose (0.5 mg) recommended in the TRANSFORMS and FREEDOMS clinical trials, taking into consideration each patient’s preferences and the potential benefits and risks of treatment [3, 4].

As per hospital protocol, all patients recommended for fingolimod treatment were referred to the SQUH ophthalmology department for assessment prior to the initiation of treatment and were thereafter routinely followed-up throughout the duration of treatment. Patients with a history of diabetes mellitus, uveitis, previous ocular surgeries, ocular and non-ocular malignancies, retinitis pigmentosa or retinal vein occlusion were not included in our study, nor were patients concomitantly taking any oral or topical medications which might induce ME [14, 15]. In addition, five pregnant women were excluded due to the potential teratogenicity of the medication, while one patient chose to discontinue the medication herself.

All remaining 21 patients, with each eye considered as an individual variable (42 eyes) underwent complete ophthalmic examinations performed by an ophthalmologist alongside basic ocular investigations, including a visual acuity test, color vision test, visual field test, and visual-evoked potential test as necessary based on a history of optic neuritis (ON). In addition, SD-OCT was performed using Zeiss Cirrus 5000 machine with macular cube of (512 × 128) mode to automatically calculate mean CFT and TMV values as compared to age- and sex-matched reference population values. Imaging was obtained for each patient prior to the initiation of fingolimod therapy at baseline, then at three, six and 12 months of follow-up for the first year. Annual SD-OCT images were taken thereafter for up to 3 years. Additional data were collected from the hospital’s electronic patient record system as required.

Collected data were analysed using the Statistical Package for the Social Sciences (SPSS) program version 28 (IBM Corp., Armonk, New York). Wilcoxon signed rank test was used to compare the paired mean values of CFT and TMV over the 4 year follow up period. A *p* value of < 0.05 was considered statistically significant.

Ethical approval for this study was obtained from the Medical Research & Ethics Committee of the College of Medicine & Health Sciences at Sultan Qaboos University (MREC approval #1946). Further authorization was obtained from the hospital information system to access patients’ medical records. Informed consent was obtained from all patients. All methods were performed in accordance with the relevant guidelines and regulations adhered to the tenets of the Declaration of Helsinki as amended in 2008.

## Results

A total of 21 patients with RRMS receiving fingolimod therapy were followed up over the four-year study period. There were five male (23.8%) and 16 female (76.2%) patients, with a mean age of 40.80 (SD = 8.17) years, 39.50 (SD = 11.39) years, respectively. In total, one male (20%) and three females (18.8%) had a history of ON. During follow-up visits, none of the patients complained of any visual symptoms including visual disturbances or loss, regardless of history of ON. Moreover, based on SD-OCT imaging, no cases of ME were detected, resulting in a FAME incidence rate of 0% in this population.

With regards to CFT, no significant differences were observed when comparing mean values obtained prior to the initiation of treatment and those obtained after starting treatment. The differences between pre-fingolimod mean CFT values and those obtained at two, three and 4 years were non-significant as shown in Tables 1 and 2. Maximum CFT readings with a mean increase of 8.375um were recorded at 3 months of follow-up, although the difference compared to baseline readings was not significant (*P* value = 0.673).

**Table 1:** Mean and median values or CFT

Variable	n	Mean ± SD	Median (IQR)
CFT - pre	32	235.81 ± 18.19	234.50 (222.25–251.00
CFT - post 3 months	8	250.00 ± 32.64	234.00 (230.00–280.75)
CFT - post 6 months	8	241.13 ± 11.90	238.00 (231.50–250.75)
CFT - post 1 year	12	228.42 ± 17.27	227.50 (213.25–242.50)
CFT - post 2 years	14	236.50 ± 16.53	235.50 (226.00–249.75)
CFT - post 3 years	24	237.92 ± 20.94	234.50 (223.75–250.00)
CFT - post 4 years	39	241.23 ± 24.91	239.00 (229.00–251.00)

Mean CFT and TMV values obtained over time using SD-OCT

*SD* Standard deviation, *IQR* Interquartile range

**Table 2:** Mean difference of paired mean values in CFT over 4 years

Time from baseline	n	Mean Difference (MD)	***P***-value*
CFT - post 3 months	8	8.375	0.673
CFT - post 6 months	6	−0.833	0.180
CFT - post 1 year	10	0.200	0.836
CFT - post 2 years	10	3.500	0.919
CFT - post 3 years	18	5.667	0.155
CFT - post 4 years	30	8.833	0.097

*Wilxon Signed Ranks test

Mean TMV values remained constant initially but showed a significant decrease towards the end of the study (Tables 3 and Table 4). The mean decrease in TMV was 0.15mm^3^ at 1 year, 0.138mm^3^ at 3 years and 0.507mm^3^ at 4 years.

**Table 3:** Mean and median values or TMV

Variable	n	Mean ± SD	Median (IQR)
TMV - pre	32	9.41 ± 0.45	9.50 (9.00–9.70)
TMV - post 3 months	8	9.31 ± 0.71	9.35 (8.60–10.00)
TMV - post 6 months	8	9.14 ± 0.37	9.15 (8.77–9.45)
TMV - post 1 year	12	9.34 ± 0.59	9.30 (8.75–9.97)
TMV - post 2 years	14	9.46 ± 0.57	9.70 (9.20–9.90)
TMV - post 3 years	24	9.36 ± 0.40	9.35 (9.20–9.70)
TMV - post 4 years	39	9.03 ± 0.68	9.20 (8.60–9.50)

*SD* Standard deviation, *IQR* Interquartile range

**Table 4:** Mean difference of paired mean values in TMV over 4 years

Time from baseline	n	Mean Difference (MD)	***P***-value*
TMV - post 3 months	8	−0.050	0.314
TMV - post 6 months	6	−0.100	0.109
TMV - post 1 year	10	−0.150	0.034**
TMV - post 2 years	10	−0.140	0.071
TMV - post 3 years	18	−0.138	0.009**
TMV - post 4 years	30	−0.507	< 0.001**

*Wilxon Signed Ranks test

**Statistically significant

## Discussion

Macular edema (ME) is a condition defined by the accumulation of fluid within the central retina or macula as a result of the breakdown of the blood-retinal barrier. There is compelling evidence supporting the role of S1P receptor activation in promoting endothelial barrier integrity [16–18]. As fingolimod causes the downregulation of the S1P receptor, it is hypothesised that this pathway is central to the development of FAME [2]. Optical coherence tomography is regarded as an effective non-invasive tool for the early detection of signs of ME, with TMV and CFT considered useful quantitative measures to track changes in the central macula over the treatment period [19].

According to the existing literature, ME is a relatively infrequent side effect of fingolimod treatment in MS patients [11]. In a 12-month study of 23 RRMS patients receiving fingolimod treatment, there was a significant decrease in mean TMV (0.065 mm^3^) at 3 months in all patients (*p* = 0.05), although this subsequently remained stable until the end of the study. Moreover, no changes in CFT were detected and none of the patients developed ME [13]. In another study, a very mild increase in mean TMV (0.025 mm^3^) was observed among 30 MS patients who were on fingolimod therapy over an average follow-up period of 5 months; however, none of the patients developed ME [12].

In general, many risk factors can lead to the development of ME in both MS and non-MS patients; accordingly, these factors should be excluded when studying the effect of fingolimod on the macula. Several ocular conditions such as; diabetic retinopathy, retinal vascular occlusion, surgical interventions, post-surgical inflammatory reactions, ocular malignancies and uveitic diseases are known to cause ME [14]. In addition, various systemic oral medications and topical eye drops can induce the development of ME as well, including oral thiazolidinediones, niacin, interferons, tamoxifen and taxanes, as well as ophthalmic eye drops containing antiglaucoma medications such as prostaglandin analogues and timolol [15].

When evaluating MS patients with suspected FAME, two differential diagnoses should be borne in mind. First, degenerative microcystic ME should be considered, a condition which presents due to neuronal loss and decreased retinal thickness secondary to the pathophysiology of MS [20]. These microcysts do not change the foveal contour and do not demonstrate vessel leakage on fundus fluorescein angiography, unlike FAME which leads to the distortion of the fovea and the collection of retinal fluid. The second differential diagnosis is uveitic inflammatory ME which may develop in patients with MS secondary to the inflammatory nature of the disease [21]. Patients with MS complicated by severe retinal periphlebitis and pars planitis may present with cystoid ME, a condition usually not found when examining a patient with FAME [22].

Overall, none of the 21 patients receiving the approved therapeutic dose of fingolimod (0.5 mg) developed ME over the course of our four-year longitudinal study. Moreover, no changes in foveal contour were detected using SD-OCT. With regards to mean CFT values, no significant changes were detected, although the highest readings were recorded 3 months after initiation of treatment. While there was a significant decrease in mean TMV values towards the end of the study (i.e., in the 3rd and 4th year), this is likely related to the degenerative loss of ganglion cells as an effect of the underlying MS, rather than the drug itself [23].

In summary, our findings confirm the relative safety, in the context of ocular side-effects, of fingolimod at a daily therapeutic dose of 0.5 mg for use in RRMS patients; in addition, the results support the need to perform ophthalmic SD-OCT imaging 3–4 months after the initiation of treatment. However, the optimal safe duration of fingolimod treatment is still questionable. A 58-year-old Caucasian male with MS was recently reported to have developed late-onset unilateral FAME after 10 years of fingolimod therapy; this was supported by fundus examination and SD-OCT imaging showing increased CFT with a foveal cyst and the accumulation of subretinal fluid, findings which resolved 3 months after discontinuation of the medication [24]. Overall, the results of our study confirm those described in previous research, although the impact of individual variations in our specific population cannot be discounted, such as disease severity, disability, age, the presence of other undiagnosed comorbidities or a history of uveitis.

## Conclusions

We sought to assess the ophthalmic effect of fingolimod therapy over a four-year period, with our findings indicating that the drug had no significant effect on the macula. These results support those reported in previous studies worldwide. Although mean TMV values decreased significantly towards the end of the study, this finding may be attributable to the disease process itself rather than the direct effect of fingolimod. Nevertheless, as previous reports have described the delayed development of FAME after continued long-term use of fingolimod (~ 10 years), further research is recommended in order to determine the optimal safe duration of treatment.

## Data Availability

The datasets supporting the conclusion of this article is included within the article and its additional files.
